# Mechanisms and Impact of Biofilms and Targeting of Biofilms Using Bioactive Compounds—A Review

**DOI:** 10.3390/medicina57080839

**Published:** 2021-08-18

**Authors:** Antony V. Samrot, Amira Abubakar Mohamed, Etel Faradjeva, Lee Si Jie, Chin Hooi Sze, Akasha Arif, Tan Chuan Sean, Emmanuel Norbert Michael, Chua Yeok Mun, Ng Xiao Qi, Pooi Ling Mok, Suresh S. Kumar

**Affiliations:** 1 School of Bioscience, Faculty of Medicine, Bioscience and Nursing, MAHSA University, Jenjarom 42610, Selangor, Malaysia; amiraabubakar19@gmail.com (A.A.M.); faradzh.etel@gmail.com (E.F.); octojie@gmail.com (L.S.J.); Hooisze0303@gmail.com (C.H.S.); akashaarif29@gmail.com (A.A.); tanchuansean@gmail.com (T.C.S.); noel0809@outlook.com (E.N.M.); yeokmun620@gmail.com (C.Y.M.); angelngxiaoqi0903@gmail.com (N.X.Q.); 2 Department of Biomedical Science, Faculty of Medicine and Health Sciences, Universiti Putra Malaysia (UPM), Serdang 43400, Selangor, Malaysia; 3 Department of Medical Microbiology and Parasitology, Faculty of Medicine and Health Sciences, Universiti Putra Malaysia (UPM), Serdang 43400, Selangor, Malaysia; 4 Department of Biotechnology, Bharath Institute of Higher Education and Research, Agharam Road Selaiyur, Chennai 600 073, Tamil Nadu, India

**Keywords:** biofilms, quorum sensing, biofouling, biofilm-related Infections, biological control, bioactive compounds, antibiofilm agents

## Abstract

Biofilms comprising aggregates of microorganisms or multicellular communities have been a major issue as they cause resistance against antimicrobial agents and biofouling. To date, numerous biofilm-forming microorganisms have been identified, which have been shown to result in major effects including biofouling and biofilm-related infections. Quorum sensing (which describes the cell communication within biofilms) plays a vital role in the regulation of biofilm formation and its virulence. As such, elucidating the various mechanisms responsible for biofilm resistance (including quorum sensing) will assist in developing strategies to inhibit and control the formation of biofilms in nature. Employing biological control measures (such as the use of bioactive compounds) in targeting biofilms is of great interest since they naturally possess antimicrobial activity among other favorable attributes and can also possibly act as potent antibiofilm agents. As an effort to re-establish the current notion and understanding of biofilms, the present review discuss the stages involved in biofilm formation, the factors contributing to its development, the effects of biofilms in various industries, and the use of various bioactive compounds and their strategies in biofilm inhibition.

## 1. Introduction

**Figure 1:**
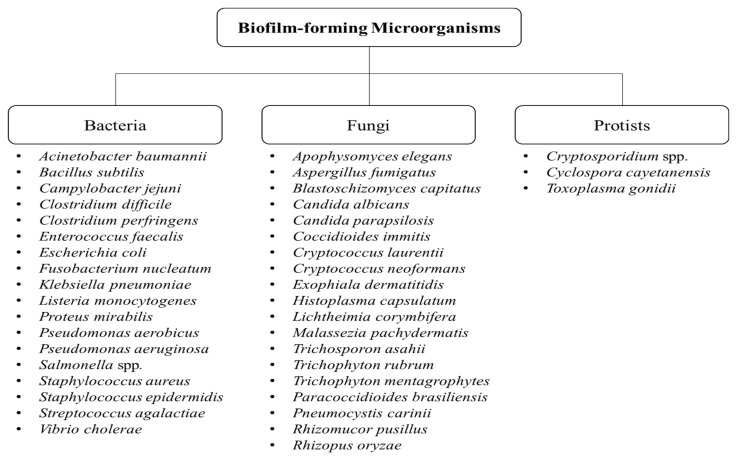
Types of biofilm-forming microorganisms [3,7,8,9,10,11,12,13,14,15,16,17].

Biofilms are defined as aggregates of microorganisms or multicellular communities that are embedded in extracellular matrix produced by the microorganisms themselves [1,2]. Some biofilms contain only a single species, while some others contain a multitude of species [3]. The matrix in which microorganisms are encased is composed of extracellular polymeric substances (EPS) typically consisting of polysaccharides, proteins or peptides, lipids, as well as deoxyribonucleic acids (DNA). These biofilm components facilitate the coherence of cells and cell surface attachment [4,5]. Biofilm formation is not only attributed to bacteria, but also to fungi and protists [3,6]. Some of the examples of biofilm-forming bacteria, fungi, and protists are shown in Figure 1.

Biofilms can be formed on any surfaces, including both biotic and abiotic surfaces [2]. Biotic surfaces such as the teeth of animals, a most common sites for bacteria to form biofilm. Biofilm formation on abiotic surfaces is prevalent in industrial and medical settings, whereby they are evidently found in industrial pipes and equipment for clinical use [2]. Among all the microorganisms, bacterial biofilm is highly associated with nosocomial diseases due to the colonization of bacteria on the surface of medical equipment, most commonly seen in indwelling urinary catheters and implantable medical devices [18,19].

Biofilm formation is the protective mechanism of microorganisms that is involved in stress coping [20]. Their survival can be prolonged in unfavourable conditions as they are able to withstand the stress when they are within biofilms. This does not happen to planktonic cells as they can easily die in such conditions [20]. The presence of any stress can trigger the formation of biofilm and cause free-floating (planktonic) bacteria to switch to the biofilm mode of growth [21]. Some common stressful conditions that are faced by the organisms include nutrient deprivation, changes in pH, and the presence of antibiotics in their surroundings, which are discussed later in this review [20].

The formation of biofilms in the human body is associated with high morbidity and mortality rates [18]. One of the reasons is because they are capable of resisting phagocytosis. Therefore, the clearing of biofilms away from the host is of great difficulty, allowing them to persist and cause chronic infections [22]. A common example is the formation of dental plaque, a type of bacterial biofilm that forms on the teeth, responsible for tooth decay and gum-related diseases [23]. Besides that, diseases caused by biofilm-forming organisms are very difficult to treat due to the organisms’ increased resistance against antibiotics. As such, it is a great challenge for researchers and physicians to search for alternative compounds or substances to target biofilms [19]. In this review, we focused on the uses of various bioactive compounds in targeting biofilm, alongside with the factors triggering and contributing to biofilm formation and its impact.

## 2. Stages in Biofilm Formation and Its Development

The development of biofilm involves four main stages, starting with cellular attachment, to formation of microcolonies, then biofilm maturation, and finally dispersion [17].

### 2.1. Cellular Attachment

Attachment of bacteria onto a surface is the initial step of biofilm formation [24]. In order for an organism to bind to a surface, they have to overcome the repulsive forces caused by the negatively charged bacterial membrane and the surface [25]. A hydrophobic surface such as plastic has a reduced force of repulsion as compared to a hydrophilic surface, for example glass and metal. The reduction in repulsive forces corresponds to increased strength of attachment [17]. Attachment is achieved by the presence of flagella and pili on the bacterial membrane [17,25]. The attachment phase involving the structural support is called a reversible attachment. The binding is reversible as bacteria are only poorly bound to a surface and are able to leave the surface at this stage. The bacteria leaving a surface return to their planktonic lifestyle [26]. For bacteria that stays on the surface, they undergo irreversible attachment, a step that is important for the transition from a planktonic to biofilm lifestyle. The adhesion of cells to a surface is aided by the surface proteins of the bacterial cells, resulting in irreversible binding [27]. As a result, the biofilm is now able to withstand stronger chemical and physical shear forces [28].

### 2.2. Microcolonies Formation

After bacterial cells have attached irreversibly onto a surface, they start to divide and produce EPS [1]. The production of EPS leads to the formation of a biofilm matrix, a ‘shelter’ where all the attached cells are living in [29]. EPS is involved in the adhesion of cells to surfaces, contributing to permanent attachment [27]. Recently, it was found that matrix proteins of a biofilm possess adhesin-like properties that mediate cellular attachment. Drescher et al. [30] showed that RbmA, one of the matrix proteins found in the *Vibrio cholerae* biofilm, acts as a mediator in this process. In addition to that, EPS mediates cellular cohesion in which bacterial cells are brought together and form microcolonies, the second step of biofilm formation [31]. Usually, a biofilm contains more than one type of micro-community and involves coordination between one community to another. This coordination is important for substrate exchange, the distribution of essential metabolic products, and also the excretion of harmful substances [17].

### 2.3. Biofilm Maturation

Cellular division with continuous production of EPS leads to the formation of an early biofilm, which becomes mature after some time and ultimately becomes a three-dimensional (3-D) structure. The aforementioned 3-D structure is contributed to by the EPS (produced by the embedded cells), and they are also responsible for maintaining this architecture [32]. The maturation process involves cell-to-cell communication, in which cells embedded in the biofilm release signaling molecules called auto-inducers to facilitate quorum sensing [17,33]. Cells that receive the signals and then express their genes coding for EPS. With the increased production of EPS, biofilms acquire the previously described 3-D structure [17,34]. Besides that, a mature biofilm also contains water channels in the matrix acting as a circulatory system. The functions of these channels include nutrient distribution and the removal of waste products [35,36].

### 2.4. Detachment of Biofilm

**Figure 2:**
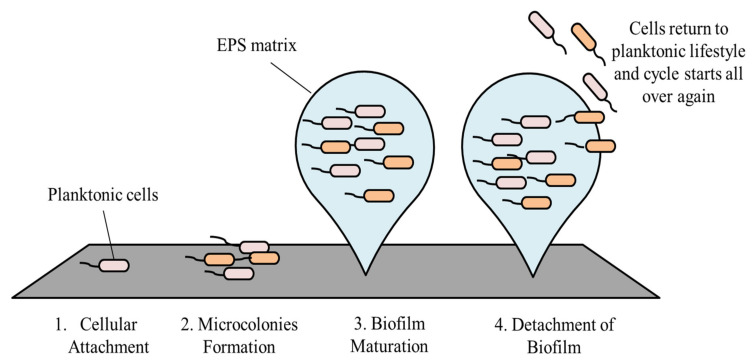
Stages in biofilm formation.

Following maturation, biofilms undergo a process known as dispersion. At this stage, some of the cells leave the biofilm and return to their planktonic lifestyle [37]. As these cells return to their free-floating form, they are now able to attach onto a new surface, and the cycle starts all over again [21]. Cells can either detach from the biofilm actively or passively. Passive dispersal of a biofilm is mediated by mechanical forces or external forces such as abrasion, fluid shear, and solid shear [38,39]. In active dispersal, it involves the upregulation and downregulation of genes. Several environmental triggers are found to be related to the dispersal of biofilms, which include nutrient starvation, insufficient oxygen supply, and changes in temperature [40]. Under these conditions, genes responsible for flagella synthesis are upregulated and this provides the bacterial cells the ability to leave the biofilm [41]. Besides that, production of dispersin B is also enhanced. This enzyme is present in the extracellular matrix and functionally acts to hydrolyze polysaccharides, resulting in EPS degradation. Increased secretion of dispersin B into the matrix negatively impacts biofilm formation and allows adherent cells to leave the biofilm easily [41,42]. Figure 2, as shown, summarizes the stages in biofilm formation.

## 3. Factors Contributing to Biofilm Formation

The ability of microorganisms to form a biofilm is dependent on the structure of the organism itself, in which organisms with pili promote the attachment and colonization of the organism to a surface. This is an initial stage that need to be completed before biofilms formation and development [43]. Flagella is another structural factor in the formation of biofilms as it gives bacteria their motility characteristic and assists in fast spreading of bacteria over the attached surfaces [44]. Apart from these structural factors, bacteria form biofilms when they detect changes that occur in the environment where they are living in. The stressful conditions threaten bacterial survival, and bacteria respond by forming a biofilm [21]. In the subsequent subsections, each of the structural and environmental factors are discussed in detail.

### 3.1. Structural Factors

#### 3.1.1. EPS

The extracellular matrix (ECM) of microorganisms is the most studied biofilm component, which plays a fundamental role in biofilm formation [45,46]. This matrix contains polymeric substances, which vary in structure and composition among different microorganisms [47]. EPS, originally referred to as ‘exopolymers’ or ‘extracellular polysaccharides’, are high-molecular-weight polymers that are synthesized by bacteria, cyanobacteria, protists, fungi, and microalgae [48,49,50,51]. The EPS consists of a vast number of organic polymers such as polysaccharides, proteins, carbohydrates, rare sugars, lipids, humic acids, and extracellular DNA [52,53].

The synthesis of EPS is a self-produced energy-demanding process attributed to the select conditions in the environment. For instance, abiotic conditions such as drought can trigger EPS biosynthesis as a response to environmental threats [54]. The EPS further affects the density, water content, charge, and mechanical stability of biofilm cells. Metaphorically referred to as ‘The House of Biofilm Cells’ by Flemming et al. [29], EPS-directed changes onto the aforesaid properties determine the biofilm cells’ condition of life in the microenvironment. This is owing to the fact that EPS biopolymers are hydrated to form a matrix that holds the cells together that allow for surface attachment, in addition to their sorption properties that enable the supply of nutrients from the environment to the biofilm organisms. The main role of EPS is to act as an impermeable barrier that offers protection against antimicrobials and environmental stressors [54]. Apart from that, the matrix structure produces nutrient gradients (alongside cell signals), resulting in the emergence of persister cells and spores, which are the highly resistant subpopulations in charge of the antimicrobial resistance mechanism [55].

Each of the EPS biopolymers play an important role in biofilms, comprising proteins as the carbon and energy reserve, polysaccharides that contribute to adhesion interactions, and extracellular DNA that is responsible for the entire formation structure [45]. Besides that, extracellular DNA (eDNA) was shown to be involved in the exchange of genetic materials between biofilm cells. A study by Hendrickx et al. [56] revealed that the amount of DNA concentration affects the frequency of transformation events whereby the events are seen to be higher in biofilms as compared to planktonic cells. A study by Roca et al. [53] reported the presence of rare sugars (fucose and rhamnose) in bacterial EPS that may play a role in providing additional biological protection to biofilms in spite of the physical barrier provided by EPS.

#### 3.1.2. Pili

Pili are important hair-like appendages that can impact the formation of biofilms in Gram-positive and Gram-negative bacteria. Several studies reinforced the aforementioned statement by comparing the biofilms of wild-type strains (with pili) and mutant strains (without pili) [15,57]. In the case of Gram-positive bacteria, Nallapareddy et al. [58] showed that the absence of pili can lead to prominent defects in biofilms formed by *Enterococcus faecalis* mutants. A study comparing wild-type and mutant strains of *Clostridium difficile* showed that the biomass and number of live cells significantly decreased in the mutant-strain biofilms. In other words, thicker masses of biofilms were observed for the wild-type strains as compared to the mutant strains [15]. Additionally, Kimura et al. [43] documented a decrease in biofilms produced by mutant strains of serotype M6 group A *Streptococcus* (GAS) (as compared to wild-type strains).

In the case of Gram-negative bacteria, researchers from China found that the absence of pili in *Salmonella* correlates with an impaired development of biofilms [57]. Furthermore, it was proven by Luo et al. [14] that pili of *Acinetobacter baumannii* is responsible for surface attachment prior to biofilm formation. This suggests that biofilms cannot be formed in the absence of pili as the aforesaid appendage is necessary for mediating the twitching motility (to spread rapidly over biofilm surfaces) of *A. baumannii* [14]. This type of motility is also found in *Pseudomonas aeruginosa*, further highlighting the significance of pili towards the foundation and development of Gram-negative-derived biofilms [59].

#### 3.1.3. Flagella

Flagella is a structure responsible for motility in bacteria, allowing them to move towards any surfaces, either living or non-living [44]. Sarah et al. [12] reported defective biofilm formation in mutated strains of *Campylobacter jejuni* (non-flagellated phenotype), accentuating the importance of flagella for effective development of biofilms. The authors demonstrated that flagella mediate the adherence of *C. jejuni* to solid surfaces, thereby initiating the formation of biofilms. They also inferred that flagella mediate cell-to-cell adhesion, correlating with the findings of Serra et al. [60]. The flagella-mediated initiation of biofilm formation was also demonstrated by Du et al. [61], using *Pseudomonas aeruginosa* as the model bacteria. They revealed that wild-type strains with flagellar motility promotes biofilm initialization in aqueous environments. This inference was proven by the higher cell counts that were recorded after incubation, as compared to the mutant strains, which conferred lower cell counts [61].

Bacterial motility is not only important for cellular attachment, but also plays an important role in biofilm maturation. The maturation of biofilms is promoted by the inhibition of bacterial motility, a process known as motility-to-sessility transition [62]. The regulation of this process involves cyclic-di-guanosine monophosphate (c-di-GMP), a small signaling molecule that resides in the cytoplasm. When the concentration of cytoplasmic c-di-GMP increases, it inhibits bacterial motility and leads to the activation of biofilm formation. Vice versa, decreased concentration of c-di-GMP does not inhibit motility and therefore biofilm formation is not activated [63]. The level of c-di-GMP determines the motility of various bacterial species (including *Pseudomonas aeruginosa* and *Escherichia coli*), ultimately affecting their ability to form matured biofilms [63].

### 3.2. Environmental Factors

#### 3.2.1. Nutritional Cues

Nutritional deprivation or low-nutrient conditions creates stress onto the microorganisms and triggers biofilm formation. However, the biofilms are not able to mature if nutrient levels are constantly low [64]. Bacterial cells present within biofilms obtain their nutrients via fluid channels that are formed in the biofilm itself. These channels not only disperse nutrients all over the biofilm, but also serves to send out toxic substances that could harm bacteria [35]. Bacteria do fail to form biofilms when they are in nutrient-rich conditions or sometimes they do form a biofilm, albeit a loose one. The bacterial biofilm formed under this condition can be easily disrupted by shear forces from fluid [26]. This is in contrast to biofilms formed under low-nutrient condition as they can withstand fluid shear force, and therefore are not easily disrupted [26]. In a study to elucidate the effects of nutrient levels towards biofilm formation (by *Bacillus subtilis*), Zhang et al. [13] found that the production of the matrix was triggered by nutrient depletion in bacterial biofilms. Reduction of carbon (glycerol and glutamate) levels in the growing medium triggers the expression of EPS promoter, resulting in increased matrix production [13].

#### 3.2.2. Oxygen Levels

Changes in oxygen levels can affect the formation of biofilms in various species of bacteria. Mashruwala et al. [65] demonstrated hypoxia-mediated biofilm formation in *Staphylococcus aureus.* Under an oxygen-limiting condition, cellular respiration is impaired, which in turn activates programmed cell death in the bacteria. As a result, the bacterial cells lyse and release proteins and DNA (components that make up the EPS) into the surroundings, suggesting that hypoxia does indeed mediate the formation of biofilms [65]. Bacterial cells in the deeper layer of the biofilm are exposed to lower levels of oxygen, and this trigger cellular apoptosis [65,66]. Consequently, more intracellular substances be released into the matrix, and this increases the strength of biofilms [67]. However, when the oxygen concentration falls to an extremely low level, cellular detachment from the biofilm can also occur [68].

Similarly, Cramton et al. [69] reported an increase in *S. aureus* biofilm formation as a result of decreased oxygen levels. Aside from inducing biofilm formation, low oxygen concentrations can also affect the maturation of biofilm. A study by Zhu et al. [70] found that depleted oxygen concentrations can impair the maturation process of biofilm, inferring that the rate of biofilm maturation is proportional to the rate of oxygen depletion. In a study by Cramton et al. [69], researchers found that polysaccharide intercellular adhesin (PIA) were also produced in higher amounts when cells are supplied with lower oxygen concentrations. PIA is a polymer produced by *S. aureus* and is used for cellular adhesion. Increased production of the aforementioned polymer facilitates cell-to-cell adhesion, an important step in biofilm formation. An in vitro study by Ghotaslou and Salahi [71] documented how hypoxic conditions can influence the gene expression in *P. aeruginosa*, causing the bacteria to produce polysaccharides extracellularly. Accumulation of these polysaccharides leads to the formation of a polysaccharide layer that makes up the biofilm.

#### 3.2.3. Temperature

The ability of bacteria to form biofilms can also be modulated by changes in temperature. The effects of temperature on biofilm production varies among different species of bacteria [72,73]. To reinforce this, the optimum temperature for biofilm production in *Salmonella* spp. was found to be 30 °C as there is a rapid planktonic-to-biofilm transition at this temperature [74]. As reported by Bonaventura et al. [7], *Listeria monocytogenes* is able to form biofilms optimally at lower temperatures (4 °C and 12 °C), which is greatly in contrast to the optimum temperature of *Salmonella* spp. In certain bacterial strains, researchers also observed an increased production of biofilms at 37 °C as compared to lower temperatures. By utilizing the crystal violet test, Hostacká et al. [8] estimated the quantitative biofilm production by *Pseudomonas aeruginosa*, *Klebsiella pneumoniae*, and *Vibrio cholerae* (non-O1 and O1) under 30 °C and 37 °C. The study demonstrated increased biofilm production at 37 °C for five of the tested strains (one strain of *P. aeruginosa*, three strains of *K. pneumoniae,* and one strain of *V. cholerae* non-O1). Mizan et al. [72] reported a significant increase in *Aeromonas hydrophila* biofilm production at temperatures between 20 °C and 25 °C. Temperatures above 25 °C and below 20 °C do not favor biofilm formation, with results showing declined biofilm production. Moreover, Obana et al. [73] found that surface adhesion can also be affected by temperature. In this study, *Clostridium perfringens* were found attached to the surface at 37 °C but not at 25 °C [73]. Table 1 summarizes the optimal temperatures for biofilm formation in different bacterial species:

#### 3.2.4. pH Levels

A change in surrounding pH levels can also contribute to the formation of biofilms. A study by Mathlouthi et al. [75] demonstrated the effect of pH on biofilm formation by *Escherichia coli* MG1655. These neutrophilic bacteria were grown on a Luria Bertani (LB) medium with pH 5.5 and pH 7.4 separately (under 25 °C and 37 °C). The results of the study showed that neutral conditions favor biofilm formation at 25 °C, with only limited formation at 37 °C. Under acidic growing environment, more biofilms are formed at 37 °C as compared to 25 °C. *E. coli* biofilms formed the best at 37 °C under acidic pH, a growing environment that resembles the host gut. From these findings, it is evident that the ability to form a biofilm is affected by pH as well as temperature [75]. Biofilm formation by *Streptococcus agalactiae* has been studied by D’Urzo et al. [11], whereby they demonstrated the promotion of biofilm formation under acidic pH. Todd–Hewitt broths with pH 7.8 and pH 5 were used and the cultures were incubated at 37 °C. Biofilms were hardly detected at neutral pH, but a significant increase in biofilm formation was detected at pH 5 [11].

#### 3.2.5. Exposure to Antimicrobials

In terms of their natural sources, antimicrobials can be found in water systems (such as rivers and lakes) at constant concentrations, caused by the continuous discharge of chemicals including antibiotics into these water sources. Microorganisms living in those affected areas are exposed to low concentrations of antibiotics for a long period of time [76]. A recent study by Salcedo et al. [77] suggested that antibiotics, with concentrations lower than that of its minimum inhibitory concentration (MIC), can mediate biofilm formation. Similar results were obtained by Strelkova et al. [78], whereby they found that any antibiotic concentrations that are below its MIC do induce the formation of biofilms. When patients affected by biofilm-forming bacteria are given antibiotics, the bacterial cells found deep in the biofilm are only exposed to very low levels of antibiotics. In this case, antibiotics have no therapeutic effects as they are unable to inhibit biofilm formation, instead they act as inducers that promote further development of biofilms [78].

## 4. Quorum Sensing in Biofilm Formation

The term quorum sensing (QS) was first described by W.C. Fuqua, denoting the cell–cell interaction of bacteria [79]. His findings were based on research from Tomasz and Nealson (as early as 1965 and 1970, respectively) who reported the discovery of autoinducer activity in *Vibrio fischeri* [80]. QS is crucial in microbial communities as bacteria can collectively obtain information about their population density, coordinate population behavior, and perform gene expression in response to variations of the surrounding population density [81]. QS is extremely beneficial for microorganisms as certain biological processes are much more expensive and unproductive for a single bacterial cell to carry out [82,83]. For instance, the execution of activities such as bioluminescence, virulence factor production, and biofilm formation require a higher population density of bacteria for maximal performance [84].

Extracellular signaling molecules known as autoinducers (AIs) govern the communication among cells through their secretion, detection, and responses [85]. The concentration of AIs is monitored by the population density of bacteria in the environment. The concentration of AIs is said to be directly proportional to the bacterial community density as the increment of bacterial population contributes to an elevation in AIs concentration [86].

The principle of the QS system in bacteria is closely dependent on the release of AIs by bacteria in a particular community. The overall mechanism of AI release is different in low cell density (LCD) and high cell density (HCD) environments [87]. As described previously, the larger the population density, the higher the concentration of secreted AIs. Hence, the concentration of AIs in HCD is comparatively higher than that in LCD, and the threshold of detection and response by receptors that are present in the bacterial membrane or cytoplasm can be attained easily. It is a circular loop denominated as a ‘feed-forward AIs loop’, which describes the detection of sufficient concentrations of AIs that enable bacteria to proceed into activation of gene expression alteration that could further promote more secretion of AI molecules [88]. This loop is most probably the key to stimulating and maintaining synchrony in bacterial populations [89].

**Figure 3:**
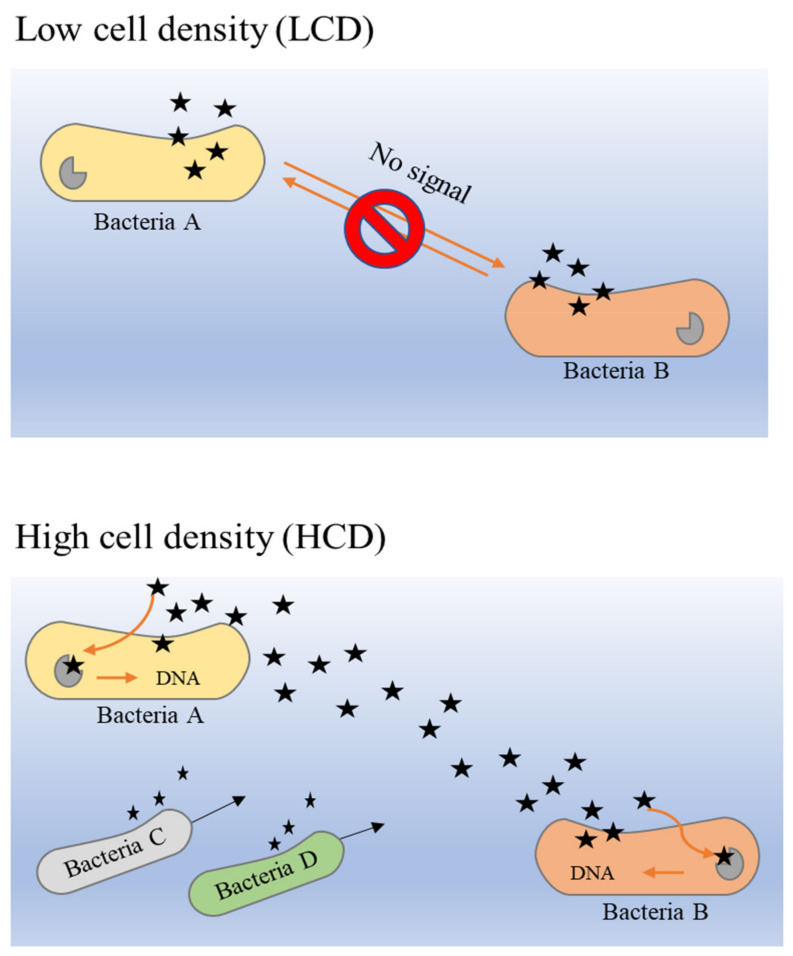
The binding mechanism of AIs molecules (bacterial QS) in LCD and HCD environments [89,90,91].

From within bacterial cells, AHLs (acyl-homoserine lactones) and AIPs (auto-inducer peptides) are produced and processed before they are secreted into the extracellular space [84]. When the extracellular concentration of AIs is elevated in HCD environments, these signaling molecules bind to the two-component histidine kinase receptor that is bound with the cognate membrane [90,91]. This activates a cascade reaction that has been activated initially through autophosphorylation to the cognate response regulator in the cytoplasm, then the activated regulator further activates transcription in the bacterial gene [89]. Figure 3 illustrates the binding mechanism of AI molecules (bacterial QS) in LCD and HCD environments.

**Figure 4:**
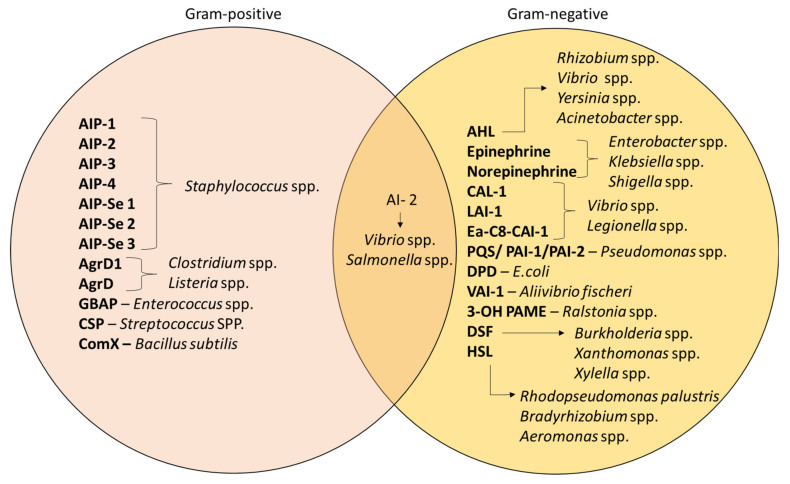
Different types of AIs in Gram-positive and Gram-negative bacteria [84,92,96,97,98].

Despite QS being recognized as a general feature of bacteria, the mechanism still differs in terms of the signaling molecules involved; namely, AI-1 and AI-2. AI-1 molecules govern intraspecies communication and it involves the acyl-homoserine lactones (AHLs)-based interaction whereas AI-2 is produced by both type of bacteria, which is the furanosyl borate diester [92]. Apart from that, studies have also differentiated the mechanism of AIs according to the types of bacteria [93]. Gram-positive bacteria such as *Staphylococcus* spp. and *Enterococcus* spp. specifically synthesize AIPs for communication [94]. *Pseudomonas* spp. and *Acinetobacter* spp. are Gram-negative bacteria that produce AHLs as their signaling molecules; the composition of AHLs consist of a lactone ring with varying lengths of aliphatic acyl chain [95]. In addition, these bacteria can also be controlled by other molecules that depend on S-adenosylmethionine as a substrate for their production [89]. Figure 4 demonstrates the different types of AIs that are released by different bacteria [84,96]. For example, *Xanthomonas* uses diffusible signal factor (DSF) [97], whereas hydroxy palmitic acid methyl ester (3-OH PAME) is used by *Ralstonia* spp. [98].

## 5. Consequences of Biofilm Formation

### 5.1. Biofouling

In various industrial fields (including water, food, medical, shipping, and oil industries), one of the most common problem of biofilm formation is biofouling [99,100]. Biofouling (also known as biological fouling) is generally defined as the unwanted growth and agglomeration of living organisms on surfaces [101]. Based on recent studies, it is now widely recognized that biofouling has negative impacts in many industries and can cost them their profitability [102]. Biofouling in medical industries only involves biofilm formation, which contrasts with biofouling in marine, manufacturing industries, and food industries as it involves a combination of biofilm formation, macrofouling, and scaling [103]. The physical and chemical properties of surfaces can influence the extent of bacterial adhesion [104]. The morphology of biofouling can be distinguished based on the thickness, bioadhesive strength, density, and type of fouling organisms [103]. For the most part, biofouling elicits a high cost in various industrial sectors due to its wide range of detrimental effects. The subsequent subsections review the aforesaid effects in more detail.

#### 5.1.1. Biofouling in Marine Industries

**Figure 5:**
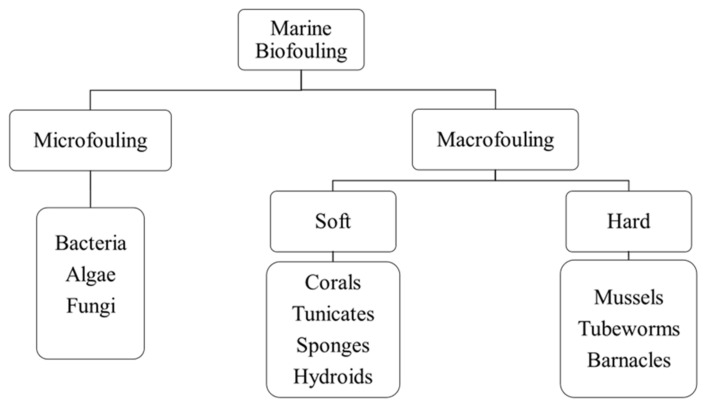
Classification of marine biofouling.

Globally, more than four thousand marine species have been associated with causing biofouling, subsequently posing a more than a serious threat in marine parks and aquacultures [105,106,107]. Marine biofouling can be categorized as microfouling (which involves the accumulation of microorganisms) and macrofouling (which involves the accumulation of macro-organisms such as invertebrates), which can further be divided into soft and hard macrofouling [104,108]. Soft macrofouling invertebrates comprise corals, tunicates, sponges, and hydroids, whereas hard macrofouling invertebrates include mussels, tubeworms, and barnacles [109,110]. These marine fouling organisms dwell better in temperate and tropical environmental conditions, but this can fluctuate according to seasonal variations [111]. According to Lebret et al. [112], marine algae is predominant among these organisms as they colonize the fastest, adhere to a broad range of surfaces, and produce numerous metabolites that have antifungal and antimicrofouling properties. Notably, some studies have described microorganisms as being involved in biofouling forms microlayers, which then initiates the adhesion of macro-organisms that can subsequently develop into macrofouling [100,104,109]. Figure 5 shows the classification of marine biofouling.

Marine biofouling appears as the visible aquatic growth of living organisms on seashore rocks, ships, boats, buoys, and underwater structures, which over time can incur physical stress on ship engines, stimulate biocorrosion of ship vessels, and interfere with mariculture environments [103,107]. A biofilm of 1 mm thickness is able to increase the friction drag of ship hulls by 80%, resulting in an overall 15% speed loss [113]. Since biofouling creates surface roughness at ship hulls, this can cause elevated ship resistance, which increases the fuel consumption (with emission of greenhouse gases), leading to high maintenance costs. To reinforce this, a study conducted by Baciocco [114] revealed that in the year 1974, the US Navy spent 200 million dollars to cover shipping maintenance and marine deterioration costs, both of which were elevated due to biofouling.

Biofouling can accelerate the decay of ship coatings due to the colonization of fouling marine species, further affecting the ship’s performance (causing unwanted noises, reduced speed, and an increase in fuel usage). This leads to an increment in unnecessary dry-docking procedures (a method of ship repair) and repainting, increasing the overall burden for more maintenance costs [107,111]. According to Munk et al. [115], ship vessel owners spend millions of dollars every three to five years just to cover the costs for dry-docking procedures, which involves the replacement of the hull coating, the cleaning of hulls, and the polishing of propellers. The accumulation of biofouling algae communities on ship walkways and structures can create a slippery-like coating, which may pose a potential safety hazard if one is not carefully onboard [103].

#### 5.1.2. Biofouling in Food and Beverage Industries

In the food industry, biofouling is prominent in processing appliances and piping systems, which gives rise to contamination, poor hygiene standards, corrosion of food equipment, food spoilage, decreased shelf-life of food products, and foodborne diseases [102,116,117]. This in turn generates functional and hygienic complications with pronounced financial losses and risks to human health [118]. These foodborne illnesses are a high risk for those who mostly consume raw food or ready-to-eat (RTE) products [119]. The severity of the diseases can range from mild gastroenteritis to life-threatening complications including liver abnormalities, meningitis, thrombotic thrombocytopenic purpura, and many others. The various environmental parameters as well as the EPS favors the persistence of biofilms in food industries, causing the overall deterioration of biofouling-susceptible appliances (pipelines, tanks, packing tools, storage materials, dispensing tubes, and pasteurizer plates) that are made of metals and alloys [120]. Sixty percent of foodborne infections were due to biofilm transfer from food-processing equipment to processed food items (which are then consumed by unsuspecting individuals) [121]. Furthermore, these surface-attached microorganisms cannot be easily eliminated as they are highly resistant to antimicrobials, ultimately contributing to severe health risks [102]. Among the various bacteria that form biofilms, *Escherichia coli* has been reported as the most tenacious foodborne pathogen commonly found in meat industries, vegetable processing industries, and preserved products [118]. Other notable bacteria that form resilient biofilms on food surfaces and are potential foodborne pathogens include *L. monocytogenes*, *S. enterica*, *C. jejuni*, *C. coli*, *S. typhimurium*, *S. enteritidis*, *B. cereus*, and *Pseudomonas* spp. [118,119,120,121,122,123,124,125,126,127,128,129,130,131,132]. Table 2 summarizes the various foodborne infections caused by biofilms in different types of food industry.

#### 5.1.3. Biofouling in Medical Industries

**Figure 6:**
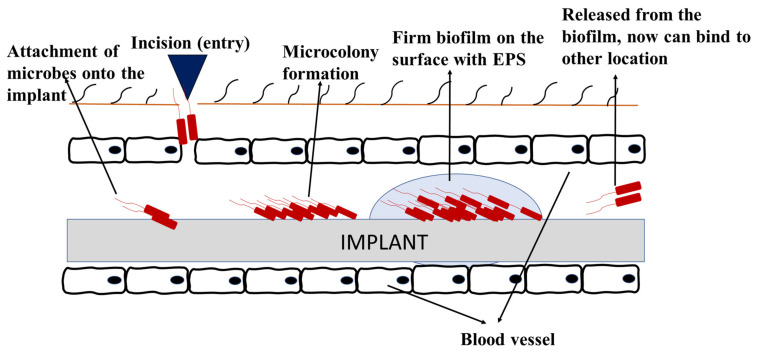
Biofouling on medical implant devices.

In medical industries, biofouling can occur in medical devices including urinary catheters, contact lenses, prosthetic implants, breast implants, dental implants, tissue fillers, cerebrospinal fluid shunts, and biosensors. As the use of these medical devices increases, the risk of biofilm infections has also increased [133]. Most of these medical devices are made of polymers, ceramics, and composites materials [18]. Biofouling in implanted devices initiates soon after the insertion of device, whereby the host-derived adhesins form a conditional layer and attracts the planktonic cells to attach onto the implant surface. Following the stages on how biofilm forms, signaling occurs, and the bacteria persists [134]. Figure 6 shows an example of how biofouling can develop on implanted medical devices. Biofilms on medical devices can arise from the patient’s skin, healthcare workers, or the surrounding environment [135]. This can cause various detrimental effects of medical biofouling, especially implant-related diseases, malfunction of devices, and implant rejection [103,136].

The malfunction of medical implanted devices results in costly surgical removal and replacement procedures [137,138]. According to Bixler and Bhushan [103], approximately 45% of nosocomial infections are attributed to implant-related diseases with over 5000 deaths per annum. The mortality rates associated with these diseases are especially high for those with contaminated cardiovascular implants i.e., prosthetic heart valves and aortic grafts [134]. In a US survey, results showed that almost 25% of blood infection-related mortality resulted from implanted vascular catheters [135]. For the most part, surgical removal is required for those implants infected by different organisms [134]. In fact, approximately two-thirds of medical implant-related infections are a result of *S. aureus* or the coagulase negative *Staphylococci* [134].

Apart from that, both the Gram-positive and Gram-negative bacteria can adhere onto medical devices and form biofilms. Some notable and well-known biofilm-forming bacteria that can cause medical implant infections include *E. coli*, *P. aeruginosa*, *C. albicans*, *K. pneumoniae*, *S. aureus*, and *S. epidermidis* [18,103,135]. An adherent biofilm can develop and lead to acute fungemia. Several studies have shown that the cells detach from a biofilm are related to mortality and cytotoxicity. Infections caused by *Candida* spp. are very common in devices like catheters. *Aspergillus* spp. are commonly attributed to infections involving cardiac pacemakers, joint replacements, breast implants, and cardiac valves. *Cryptococcus neoformans* can also colonize devices similar to the aforementioned examples and cause severe infections [139].

### 5.2. Biofilm-Related Infections

Approximately 65% of infections that are caused by bacteria are associated with biofilms [17]. Some of the common non-device-related biofilm infections are discussed in the following subsections.

#### 5.2.1. Periodontitis

Periodontitis is an infection of the gums whereby the soft tissues and the bones supporting the teeth gets damaged as a result of the infection. This could develop due to poor oral hygiene, which may also result in tooth loss. The infectious agents behind this infection are *Pseudomonas aerobicus* and *Fusobacterium nucleatum* [17]. These bacteria can form biofilms in the mucosal surfaces of the mouth. Through this way, they can invade the cells, release their toxins, and form plaques in a couple of weeks [17]. Moreover, the formation of lesions may be present due to the infection. The result of the treatment may be influenced by the size of the lesion [140]. Antimicrobial treatment is enough in the case of minor infections, but special treatment may be required when the infection becomes severe [141].

#### 5.2.2. Rhinosinusitis

The inflammation of the paranasal sinuses is referred to as sinusitis or rhinosinusitis. Depending on the severity of the infection, it can be classified as acute or chronic. It is said that most of this infection is caused by the colonization of bacteria. However, some studies have also stated that fungal biofilms might also be involved. Both *Aspergillus fumigatus* and *Staphylococcus aureus* have been discovered to be involved in causing rhinosinusitis [142]. It has also been stated that the patients that have undergone surgical procedures are more likely to be infected with the infection related to biofilms. Biofilms are formed in the presence of more eosinophil cells. Moreover, bacteria such as *P. aeruginosa*, *S. pneumoniae*, *S. aureus*, and *H. influenza* are also involved in causing chronic rhinosinusitis [143]. The most dominant species is *S. aureus,* which was proven in a study conducted by Schurmann et al. [144]. It was further concluded that most of the chronic rhinosinusitis infections are associated with microbiomes comprising of multiple different species.

#### 5.2.3. Cystic Fibrosis (CF)

Cystic fibrosis is an autosomal recessive disease caused by a mutation in the gene coding for the cystic fibrosis transmembrane conductance regulator (CFTR) protein. The most affected areas are the gut and pancreas in which there is a failure to remove the mucous secretions. It mainly involves constant coughing, and the lungs tend to be more prone to infections [145]. Almost 80% of infections seen in cystic fibrosis are related to biofilms. The lung environment of a CF patient is favorable to *P. aeruginosa* and that is why the bacterium is able to dominate in the airways. Their resistance to antibiotics occurs due to increased production of EPS [146]. Studies have shown that the presence of the microbial agent leads to poor prognosis in CF patients. *P. aeruginosa* is able to form drug-resistant biofilms, which makes antibacterial therapy useless [147,148].

## 6. Use of Bioactive Compounds as Antibiofilm Agents

**Figure 7:**
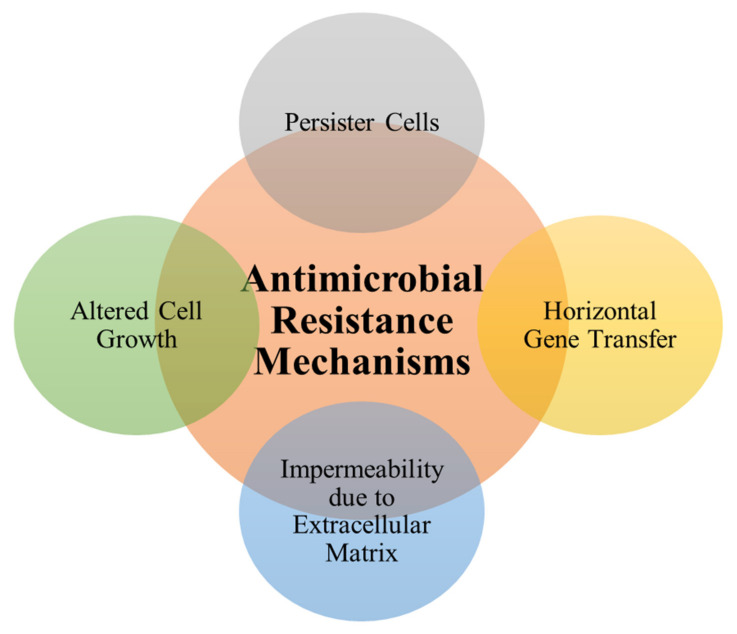
Mechanisms of antimicrobial resistance in biofilms.

The vital principle in developing strategies to control biofilms is by first understanding the mechanisms involved in biofilm resistance [149,150]. Biofilms are difficult to control due to their ‘resistant’ nature that can develop from the attachment phase and increase as they age (varies among different microorganisms) [151,152,153]. More so, the antimicrobial resistance in biofilms is 100 to 1000 times more than their correspondent planktonic cell forms [154]. There are various mechanisms responsible for antimicrobial resistance in biofilms. Donlan [155] inferred in his study the different means by which biofilm develop resistance and these included the changes in cellular growth rate, whereby the growth of sessile cells is much slower than the planktonic cells during formation, and this produces the dormant cells, which recede the antimicrobial uptakes. He added the relative impermeability of antimicrobial molecules due to the matrix materials; acting as a barrier which results in antimicrobial hinderance. A good example is in CF patients, in which *P. aeruginosa* overproduces EPS and forms a sticky mucoid biofilm that protects the cells from opsonization by host antibodies and also prevents the diffusion of antimicrobials to the target cells. Other factors may include the phenomenon of persister cells, which form a subpopulation of persistent cells that are multi-drug resistant [153]. For instance, *Candida albicans* predominant in oral thrush biofilms have high persister (*hip*) mutants that resists antimicrobial therapies [156]. In addition, Del Pozo and Patel [149] stated that horizontal gene transfer plays a role in multidrug resistance within biofilms, and they also described how stress-response genes may be upregulated (forming resistant phenotypes) due to changes in environmental conditions (such as nutrients, oxygen, pH, and temperature). To illustrate, *Pseudomonas aeruginosa* develops antimicrobial resistance to cationic antimicrobials e.g., Polymyxin B (PXB) due to nutritional loss of magnesium ions as well as β–lactam resistance in *Escherichia coli* due to iron limitations [157]. Figure 7 summarizes the four main mechanisms that are involved in antimicrobial resistance in biofilms.

The conventional physical and chemical methods applied in treating biofilms have been rendered ineffective and result in environmental pollution. There is a need for new strategies against biofilm due to their increased resistance against antimicrobials and host immune system [158]. Biological control of biofilms employs certain mechanisms (from living matter, microorganisms, or microbes within the biofilm itself) in order to interfere with their existence [118]. This strategy is mainly to target the QS, degrade the extracellular matrix, inhibit microbial adherence, and eliminate persister cells. Various natural compounds are of great interest in drug discovery due to their enormous advantages and are derived from plants, fungi, bacteria, and other animals [159]. Table 3 summarizes the types of bioactive compounds, sources, target bacteria, and their respective strategies in inhibiting biofilm formation. The subsequent subsections focus on the use of non-toxic and natural antibiofilm agents as a promising biological control method for biofilm inhibition.

### 6.1. Plant-Derived Bioactive Compounds

#### 6.1.1. Phenolics and Polyphenolics

##### Flavonoids

Flavonoids are phenolic compounds (predominant in photosynthesizing cells) that are mostly found in fruits, vegetables, nuts, flowers, wine, and tea [159,160]. Their basic structure comprises two benzene rings linked by three carbon atoms, which forms an oxygenated heterocyclic ring. Flavonoids are further divided into sub-classes based on their type of heterocyclic ring, including isoflavonoids, 3-phenyl-benzopyrans, neoflavonoids, and 4-phenyl-benzopyrans [161]. It is well-established and evidently documented that flavonoids exhibit various bioactive effects such as antibacterial, antifungal, antiviral, and antiprotozoal activities [162,163]. They have been utilized as therapeutics used to treat a number of diseases [160]. It is known that their activity is a result of their capability to bind with extracellular and soluble proteins, which increases their permeability to bind to bacterial cell walls [159]. In fact, those with high lipophilic content also disrupt bacterial membranes. Kaempferol and naringenin (flavonoids present in citrus plant) are effective as QS inhibitors against *Escherichia coli* O157:H7 and *Vibrio harveyi* BB120 by interfering with AHLs and their receptors [164,165]. Another possible mechanism of biofilm inhibition by flavonoids is reported by Valsaraj [166], whereby 7-hydroxy-3,4-(methylenedioxy) flavan derived from the fruit peel of *Terminalia bellirica* had an antifungal property against *Candida albicans* by interfering with their metabolism. Catechin derived from green tea has also exhibited antibacterial activity that interfere with biofilm formation of *Porphyromonas gingivalis* by forming complexes with bacterial cell walls of the microorganisms [167]. Phloretins that are isolated from apples have been reported to inhibit biofilm-forming *E. coli* O157:H7 without inhibiting planktonic cells growth [168].

##### Tannins

Tannins are one of the major phytochemicals distributed in various parts of plants such as bark, wood, leaves, fruits, and roots [159]. The pharmacological effect of tannins lies in the type of plant, evidently utilized as antibacterial, antiviral, antioxidant, and antihelminth remedies [169]. Based on their physiochemical properties, tannins are divided into either hydrolysable or condensed types. Antibacterial properties of tannins have been described as bactericidal and bacteriostatic against common pathogens including *E. coli*, *Salmonella* spp., *Pseudomonas* spp., *Streptococcus* spp., and *Staphylococcus* spp. [159]. Tannins primarily inhibit bacteria by forming complexes with their polysaccharides. In addition, the mechanism of tannins in biofilm inhibition is the inactivation of microbial adhesins, enzymes, and membrane proteins [159]. A study by Lee et al. [170] found that tannic acids (TAs) can inhibit biofilm formation of *S. aureus* by suppressing the genes (*agrA*, *icaA*, and *icaD*) that are responsible for bacterial adhesion. Hamamelitannins (isolated from *Hamamelis virginiana* leaves) were also found to have antibiofilm activity against medical implanted device-related infections through QS regulator RNAIII inhibition [171].

##### Phenolic Acids

Phenolic or phenol carboxylic acids are found in a variety of plant-based foods especially seeds, fruit peels, and vegetable leaves, which consist of the highest amounts. They can be classified into hydroxycinnamic acids (caffeic and sinapic acids) and hydroxybenzoic acids (vanillic and syringic acids). Phenolic acids have diverse biomedical uses including antioxidant, anticancer, antimicrobial, and anti-inflammatory [172]. The potential of bacterial inhibition is based on the number and position of the hydroxyl groups on their aromatic rings [159]. The antibacterial activity of phenolic acids is due to their capability to inhibit nucleic acid synthesis, inhibit bacterial enzymes, and penetrate cytoplasmic membranes of bacteria [159]. Research by Sánchez-Maldonado et al. [173] discussed the use of hydroxycinnamic and hydroxybenzoic acids as an effective antibacterial against lactic acid bacteria, *L.fermentum*, *L.plantaruma*, and *L.brevis* in which their activity was dependent on number of hydroxyl groups. Besides that, gallic and ferulic acids were also shown to be effective against *P. aeruginosa*, *E. coli*, *L. monocytogenes*, and methicillin-resistant *Staphylococcus aureus* (MRSA) [173].

##### Coumarins

Coumarins (naturally found in numerous plants) are volatile active compounds, which comprise fused benzene and pyrone rings [174]. These compounds have many important bioactive properties, including antimicrobial, analgesic, and anti-inflammatory activities [175]. Some common coumarins (including esculetin, scopoletin, coladonin, psoralen, umbelliferone) have been reported to inhibit the formation of *P. aeruginosa*. Moreover, umbelliferones were found to display antibiofilm activity against *E. coli* 0157:H7 but they do not inhibit planktonic growth [176]. Furocoumarins from grape juice could inhibit autoinducer-1 and autoinducer-2 signaling against TN5 mutants of *Vibrio harveyi* and they are also able to repress *E. coli* biofilm formation through QS inhibition [177]. Some of the coumarins have also been reported to have inhibitory effects against *Candida albicans* and can be used to cure vaginal candidiasis [174].

#### 6.1.2. Alkaloids

Alkaloids are heterocyclic nitrogen compounds produced by several plants. The first clinically relevant alkaloid was discovered in 1805 from the opium *P. somniferum,* which was widely applied in treating medical conditions [159,174]. Berberine isolated from the roots and stems of berberis has been used as a folk medicine due to its antibacterial, antifungal, antiviral, and antiprotozoal properties. It is known to target the RNA polymerases and nucleic acids of several microorganisms [178,179]. Indole alkaloids derived from ethanolic extracts of *Terminalia chebula* was proven to be an effective antibacterial against most common multi-drug resistant (MDR) strains such as *P. aeruginosa*, *E. coli*, *S. aureus*, *A. tumefaciens*, and *B. subtilis*. [180].

#### 6.1.3. Terpenoids and Essential Oils

Terpenoids or terpenes are among the largest groups of phytochemicals, which can be further divided to monoterpenoids, diterpenoids, and sesquiterpenoids, among others based on their carbon building blocks. They are the main class of the constituents of essential oils (EO), which are reported frequently over the years due to their diverse bioactivities [159]. These EOs (when comprised with oxygen synthesized from acetate units) make up the terpenoids, which share a common origin with fatty acids [174]. Their mechanism against biofilms is not fully understood but some studies have postulated that it is a result of membrane disruption by the lipophilic components. This in turn elevates membrane permeability and change of ion transport processes in both Gram-negative and Gram-positive bacteria [159,160]. Thymoquinone, a constituent of black seed oil isolated from *Nigella sativa,* is more inclined towards Gram-positive biofilm inhibition of *S. aureus* and *L. monocytogenes* as compared to Gram-negative biofilms. This is discussed in a similar study done by Szczepanski and Lipski [181] that have reported no inhibition of Gram-negative proteobacteria such as *S. enterica* and *P. aeruginosa* by EOs. Gossypols derived from cotton seeds have been reported to inhibit Gram-negative bacteria including *P. vulgaris*, *E. coli*, and *P. aeruginosa* as well as several Gram-positive bacteria including *S. epidermidis, B. subtilis*, and *B. cereus* [159].

#### 6.1.4. Lectins

Lectins are carbohydrate-binding proteins comprising of disulfide bonds occurring in multiple plants tissues such as the leaves, barks, bulbs, fruits, and flowers. Their mechanism is attributed to the formation of ion channels in mitochondrial membrane [174]. The families of lectins include the legume, chitin-binding, mannose-binding, and jacalin-related lectins, which do not share a common structure but rather differ in structures, sizes, molecular organization, and active sites although having the same activity of binding to carbohydrates promoting antibacterial effects [159]. Their mechanism of action is through the interaction with bacterial cellular wall components including peptidoglycans, lipopolysaccharides (LPS), teichoic, and teichuronic acids [182,183,184]. When they interact with bacteria, lectins affect their adherence and therefore interferes with biofilm formation (as well as planktonic growth). Lectins extracted from the seaweed *Solieria filiformis* have been reported to reduce the growth of planktonic cells of *P. aeruginosa* as well as other Gram-negative species. Moreover, lectins can also be isolated from algae and show similar results. To reinforce, lectins derived from the red algae *B. triquetrum* are able to inhibit *Streptococcus* spp. through attachment to their pellicle [185]. Furthermore, some lectins are non-selective in their inhibitory effects towards Gram-positive and Gram-negative bacteria including *E. coli*, *S. aureus*, *B. subtilis*, *P. aeruginosa*, and *Klebsiella* spp. Some researchers have reported that lectins isolated from *Myracrodruon urundeuva* had increased specificity for N-acetylglucosamine, indicating more antibacterial effects against Gram-positive than Gram-negative bacteria [186].

#### 6.1.5. Peptides

Antimicrobial peptides (AMPs) are short (15 to 30 amino acids) and positively charged peptides that are present in all living organisms [159,187]. With regard to their size, primary structure, and cysteine content, plant AMPs can be divided into distinct families such as thionins, lipid transfer proteins, defensins, snakins, hevein, and knottin-like proteins [188,189,190]. Many AMPs exhibit a wide range of antimicrobial activity against bacteria, fungi, viruses, and protozoa. They display strong antibiofilm activity against the MDR strains as well as various clinically isolated pathogens [191].

Their mechanism of action mainly involves the interaction with membranes of bacteria, disruption of membrane, and intracellular targets within bacterial cells [187]. AMPs target the intracellular structures, which result in the alteration of metabolic processes, including protein synthesis and cytosolic enzyme activity inhibition [192,193]. In order to penetrate bacterial membranes, the length, hydrophobicity, charge, and amphipathic structure of AMPs are taken into consideration [194,195]. Since AMPs are mostly cationic, they interact with negatively charged components of bacteria, including lipopolysaccharides and teichoic acids. Most membrane-active AMPs that eliminate planktonic growth also have effect on biofilm formation at low concentrations. The partial replacement of L form to D form amino acids is known to increase antibacterial activity against planktonic cells since it tends to promote peptide resistance against protease degradation and reduce hemolytic activity [196]. This is evident in the inhibition of biofilms in every stage throughout their development cycle. There is great attention paid to the use of AMPs (such as LL-37) as therapeutic agents due to their capability to inhibit biofilm formation [197].

A recent study has shown that RsAFP2 plant defensins (isolated from the seeds of *Raphanus sativus*) are effective against *C. albicans* biofilm formation. The same study found that TnAFP1 (from *Trapa natans*, water chestnut) can also inhibit *C. tropicalis* biofilm formation in a concentration-dependent manner [198]. Circulins A-B (derived from *Chassalia parviflora*) and cyclopsychotride A (from *Psychotria longipes*) were documented to have antimicrobial activity against many common pathogenic bacteria such as *Klebsiella* spp., *E. coli*, *S. aureus*, *P. vulgaris*, and *M. luteus* [199,200,201].

### 6.2. Animal-Derived Bioactive Compounds

#### 6.2.1. Chitosan

Chitosans are polyaminosacchrides derived from the deacetylation of chitin, which naturally occurs in the exoskeleton of shellfish such as crab, lobster, shrimp, and prawn [202]. Owing to its biocompatibility and biodegradability properties, chitosan is known for its antimicrobial properties (bacteriostatic and bactericidal effects) against the majority of bacteria. More so, it exhibits antibiofilm activity through several mechanisms such as the inhibition of protein synthesis, binding to the bacterial cell wall, and the suppression of bacterial growth by external barrier formation [203,204]. The cationic nature of chitosan allows it to penetrate and interact with the negatively charged cell membrane surface, subsequently interfering with biofilm formation [203]. As explained by Goy et al. [205], the inhibition of protein synthesis occurs through the penetration of chitosan into the nuclei of microorganisms where these chitosan oligomers can then be observed inside the bacteria through a confocal laser scanning microscope. The mode of suppression of spore elements and binding to vital nutrients help inhibit the bacterial growth as reported. The extent of chitosan biological activity depends on the molecular weight and the degree of acetylation [205]. To illustrate, studies conducted with differences in molecular weight on *E. coli*, *S. aureus*, *S. enterica*, *B. subtilis*, *B. cereus*, and *K. pneumoniae* showed that a lower chitosan molecular weight resulted in increased growth inhibition. Likewise, a lower degree of acetylation resulted in increased antimicrobial activity [205]. Research by Orgaz and coworkers [206] was done to exploit the effectiveness of chitosan against four mature biofilms and their planktonic cells including *S. enterica*, *S. aureus*, *L. monocytogenes*, and *B. cereus*. The results showed higher susceptibility for the biofilm cells as compared to planktonic cells except for *S. aureus*. Chitosan and its derivatives have been applied in the protection of implanted medical devices (made of pure titanium) against biofilm-forming microorganisms, in which *S. epidermidis* and *S. aureus* have shown complete shrinkage of the bacterial cells [207]. Some studies have also proposed the use of chitosan as a carrier for antibiofilm drugs since it can enable the effective, prolonged, and controlled release of the aforesaid drugs [207].

#### 6.2.2. Hyaluronic Acid

Hyaluronic acid (HA) is a glycosaminoglycan consisting of glucuronic and N-acetylglucosamine disaccharide blocks that are mostly abundant in skin and connective tissues [208]. Based on its concentration and molecular weight, HA has the ability to interrupt bacterial adherence and biofilm formation. A study to report the biofilm activity of hyaluronic acid was done against *S. epidermidis* whereby it was able to disrupt the bacterial adherence and exhibited antifouling properties [208]. Another study reported that the concentration of HA greatly influences its biofilm activity and results inferred *S. aureus* biofilm to be sensitive against HA action as compared to other respiratory tract pathogens [209]. Although there is not much research done on hyaluronic acid, HA is utilized for the coating of implanted medical devices that acts as a protective barrier against biofilm-related diseases, thereby reducing the chances of biofouling. In addition to that, some studies have reported the bacteriostatic effects of HA against various oral cavity pathogens [210,211].

## 7. Conclusions and Future Perspectives

There is a crucial need for developing new therapeutic strategies that can be effective against biofilm-related infections as well as biofouling in industries. The extent of biofilm-associated effects result in enormous costs to society, approximately billions of dollars per annum, and in severe cases, the related infections may lead to death. Since then, the conventional methods used have been rendered ineffective due to the increased multi-drug resistance of microorganisms. Understanding the mechanisms involved in MDR assists in developing compounds to specifically target those mechanisms and thus prevent and control biofilm formation in general. The use of plant- and animal-derived bioactive compounds have shown to be effective against Gram-positive and Gram-negative bacteria as well as other biofilm organisms, which is considered to be safer as compared to physical and chemical control methods. In the future, we hope that these bioactive compounds can be translated into potential antibiofilm drugs, which could act as promising models in combatting the various biofilm/biofouling organisms.

## Figures and Tables

**Table 1:** Optimal temperatures for biofilm formation in different bacterial species.

Type of Bacteria	Optimum Temperature	References
*Salmonella* spp.	30 °C	[74]
*Listeria monocytogenes*	37 °C	[7]
*Aeromonas hydrophila*	20–25 °C	[72]
*Clostridium perfringens*	37 °C	[73]

**Table 2:** Effects of biofouling in food and beverage industries.

Type of Food Industry	Prominent Bacteria	Effects	References
Dairy Industry	*L. monocytogenes* *S*. *typhimurium* and *S*. *enteritidis* *E. coli* (STEC) *B. cereus*	Gastroenteritis or listeriosis Gastroenteritis Enterohemorrhagic gastroenteritis or hemolytic uremic syndrome (HUS) Gastroenteritis or occasionally acute liver failure	[120,122,126,129,131]
Poultry Industry	*S. enterica* *C. jejuni* and *C. coli*	Gastroenteritis or septicemia Enterocolitis or gastroenteritis	[120,123,128]
Meat Industry	*E. coli* O157:H7 *L. Monocytogenes* *Salmonella* spp.	Hemorrhagic colitis or thrombotic thrombocytopenic purpura (TTP) Gastroenteritis or listeriosis Salmonellosis	[118,124,127,130,132]
Fish and Seafood Industry	*Vibrio cholerae* *Aeromonas* spp. *Pseudomonas* spp.	Cholera or gastroenteritis Epizootic ulcerative syndrome (EUS)	[119,121,122,125]

**Table 3:** Types of bioactive compounds and their strategies in biofilm inhibition.

Type of Compound	Class/Subclass	Source	Target Microorganism	Antibiofilm Strategy	References
Flavonoids	Kaempferol and Naringenin	Citrus plants	*E. coli* O157:H7,	QS inhibition by interfering with AHL and their receptors	[164,165]
			*V. harveyi* BB120		
	7-hydroxy-3,4-(methylenedioxy) flavan	*Terminalia bellirica* fruits	*C. albicans*	Metabolism inhibition	[166]
	Catechin	Green tea	*P. gingivalis*	Forms complexes with bacterial cell walls of microorganisms	[167]
	Phloretin	Apple	*E. coli* O157:H7	-	[168]
Tannins	Tannic acid	Tea	*S. aureus*	Suppression of QS genes involved	[170]
	Hamamelitannin	*Hamamelis virginiana* leaves	*-*	QS regulator RNAIII inhibition	[171]
Phenolic Acids	Hydroxycinnamic and hydroxybenzoic acids	-	*L. fermentum*, *L. plantaruma, L. brevi*	Dependent on number of hydroxyl groups	[173]
	Gallic and ferulic acids	-	*P. aeruginosa*, *E. coli*, *L. monocytogenes,* methicillin-resistant *Staphylococcus aureus* (MRSA)	-	[173]
Coumarins	Umbelliferone	-	*E. coli* O157:H7	-	[176]
	Furocoumarins	Grape juice	*Vibrio harveyi, E. coli* O157:H7	Inhibition of QS molecules, AI-1 and AI-2	[177]
Alkaloids	Indole alkaloids	*Terminalia chebula*	*P. aeruginosa*, *E. coli*, *S. aureus*, *A. tumeficaens, B. subtilis,*	-	[180]
	Berberine	*Berberis* roots and stems	-	Target the RNA polymerases and nucleic acids of microorganisms	[178,179]
Terpenoids and Essential Oils	Thymoquinone	*Nigella sativa*	*S. aureus, L. monocytogenes*	-	[181]
	Gossypols	Cotton seeds	*P. vulgaris, E. coli, P. aeruginosa, S. epidermidis, B. subtilis, B. cereus*	-	[159]
Lectins	-	*Solieria filiformis*	*P. aeruginosa*	-	[185]
	-	*B. triquetrum*	*Streptococcus* spp.	Attachment to the pellicle	[185]
Peptides	RsAFP2	*Rhapanus sativus* seeds	*C. albicans*	-	[198]
	TnAFP1	*Trapanatan* fruits	*C. tropicalis*		
Peptides	Circulins A-B	*Chassalia parviflora*	*Klebsiella *spp., *E. coli*, *S. aureus*, *P. vulgaris*, *M. luteus*	-	[199,200,201]
	Cyclopsychotride A	*Psychotria longipes*			
Chitosan	Chitin	Shell of shrimp, lobster, crab and prawns	*S. enterica*, *S. aureus*, *S. epidermidis*, *L. monocytogenes, B. cereus*.	Inhibition of the protein synthesis, binding to the bacterial cell wall and suppression of bacterial growth by external barrier formation	[203,204,206]
Hyaluronic Acid	-	-	*S. epidermidis*	Inhibition of microbial adherence	[208]

## Data Availability

Not applicable.
